# MidMedPol: Polychaetes from midlittoral rocky shores in Greece and Italy (Mediterranean Sea)

**DOI:** 10.3897/BDJ.1.e961

**Published:** 2013-09-16

**Authors:** Kleoniki Keklikoglou, Sarah Faulwetter, Giorgos Chatzigeorgiou, Fabio Badalamenti, Militiadis Spyridon Kitsos, Christos Arvanitidis

**Affiliations:** † Biology Department, University of Crete, Vasilika Vouton, Heraklion Crete, Greece; ‡ Hellenic Centre for Marine Research (HCMR), Gournes, Heraklion Crete, Greece; § National and Kapodestrian University of Athens, Athens, Greece; | CNR-IAMC Laboratorio di Biologia Marina, Castellammare del Golfo, Italy; ¶ Department of Zoology, School of Biology, Aristotle Univeristy of Thessaloniki, Thessaloniki, Greece

**Keywords:** Midlittoral zone, Polychaeta, rocky shores, Mediterranean Sea, biodiversity, intertidal, Italy, Greece, 1984–2009

## Abstract

This paper describes a dataset of polychaetes (Annelida) from 14 midlittoral rocky shore sampling sites in Greece and Italy (Mediterranean Sea). The dataset combines the outcome of four different projects studying the hard substrate midlittoral zone in the Mediterranean between 1984 and 2009. Samples were collected by scraping and collecting the organisms from a framed area. The maximal sampling depth was 1.5 m. In total, 123 polychaete species were recorded, five of which are new records for the respective biogeographic sectors of the Mediterranean. The dataset contains 788 occurrence records, fully annotated with all required metadata. These data contribute to the knowledge of a previously very understudied regional habitat, since at present, comprehensive lists of the midlittoral communities in the Mediterranean are provided through only a few, paper-based, studies. This dataset is one of the first electronic data compilations of the Mediterranean midlittoral zone communities and certainly the most comprehensive of its kind, contributing to the ongoing efforts of the Ocean Biogeographic Information System (OBIS) which aims at filling the gaps in our current knowledge of the world's oceans. It is accessible at http://ipt.vliz.be/resource.do?r=mediterraneanpolychaetaintertidal.

## Introduction

The Mediterranean Sea is an enclosed water basin with a very low tidal range, in the range of 20–40 cm (Day et al. 1995). Its intertidal zone is accordingly very narrow, and is often referred to as "midlittoral zone" instead of "intertidal zone", following the terminology of Stephenson and Stephenson (1949). Pérès and Picard (1964) subsequently described the hard bottom biocoenoses of the midlittoral zone in the Mediterranean Sea and defined its ecological attributes by using characteristic species. The midlittoral zone can also be created by considerable and steady wave-action without the existence of true tides (Stephenson and Stephenson 1949). Such irregular rhythms of immersion/ desiccation which depend on weather conditions create an extreme environment, allowing only species with certain characteristics to survive.

Despite the ecological importance and easy accessibility from the shore, only few studies have examined the species communities of the Mediterranean midlittoral zone (e.g. Ben-Eliahu and Safriel 1982, Cardell and Gili 1988, Sardà 1991). Most of these studies are paper-based and the information contained within is not readily accessible in machine-readable formats. Electronically available biogeographic information for the Mediterranean Sea is still fragmented for all subregions and habitats (Arvanitidis et al. 2006), and none of the global biogeographic databases (OBIS, http://www.iobis.org; GBIF, http://data.gbif.org) contain systematically collected data on the Mediterranean midlittoral zone.

This study attempts to increase our current knowledge of the rocky midlittoral zone of Mediterranean Sea by providing species occurrence data of polychaete species, assembled from four independent and previously unpublished datasets. Polychaetes are often used as a representative group of macrobenthic communities because they tend to be the dominant taxon in these communities and hence, they are used as indicators of environmental disturbance (e.g. Giangrande et al. 2005, Olsgard et al. 2003). The present dataset contains georeferenced and fully documented information on 123 species (788 individuals) of polychaetes, recorded from 14 regions/ sampling sites in the Aegean Sea and in Italy, from 1984 to 2009 (Table 1). Five species are new records for the respective biogeographic sectors in the Mediterranean region.

## Project description

### Title

This dataset combines the data of four independent sampling campaigns: (a) the monitoring of midlittoral rocky shores in Crete in the framework of the NaGISA project (Natural Geography in Shore Areas, http://www.coml.org/projects/natural-geography-shore-areas-nagisa); (b) the study of the biodiversity of midlittoral rocky shores in the framework of the PhD thesis of Militadis-Spyridonas Kitsos (Aristotelian University of Thessaloniki); (c) the preliminary study to establish marine protected areas in Sicily (Capo Gallo and Zingaro, north-western coast of Sicily, Italy) and (d) a monitoring project to assess the effects of a temporal explosion of *Sabellaria* spp. and *Mytilaster* spp. (Balestrate, north-western coast of Sicily, Italy).

### Personnel

Christos Arvanitidis, HCMR (project coordinator, sample collection), Georgios Chatzigeorgiou, HCMR (sample collection, sample identification), Sarah Faulwetter, HCMR (sample collection, sample identification, data management), Kleoniki Keklikoglou, HCMR/University of Crete (sample identification, data management), Fabio Badalamenti, CRN-IAMC, Italy (sample collection, sample identification), Militadis-Spyridonas Kitsos, Aristotle University of Thessaloniki (sample collection, sample identification), Lennert Tyberghein, VLIZ (data management), Wanda Plaiti, HCMR (sample collection), Vasiliki Markantonatou, HCMR (sample collection), Ioannis Pesmatzoglou, HCMR (sample collection), Rick Fernandez and students from Niceville High School, FL, USA (sample collection), Kalliopi Ousantzopoulou and students from Heraklion High School of Arts, Crete, Greece (sample collection).

### Study area description

**Figure 1.**
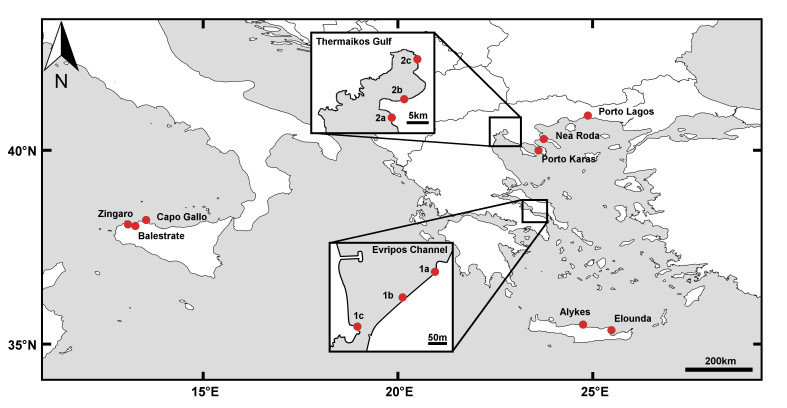
Map of the sampling locations

This dataset includes records from 14 sampling sites at 10 different locations: Alykes, Elounda, Evripos channel, Thermaikos Gulf, Nea Roda, Porto Karas and Porto Lagos in Greece and Balestrate, Zingaro and Capo Gallo in Italy (Table 1, Fig. 1).

**Alykes and Elounda:** Both sampling sites are located on the North coast of Crete (Eastern Mediterranean) and are characterised by a continuous hard bottom habitat with dense algal coverage (*Cystoseira* spp., *Sargassum* sp., *Corallinales* spp.) and a moderate wave exposure. The area of Alykes has on average a denser algal coverage than the area of Elounda. The intertidal substrate is dominated by limestone rocks. None of the sites is impacted by detectable anthropogenic activity, though a sandy beach in ca 500 m distance of the sampling area in Elounda is subjected to moderate beach tourism and increased leisure boat traffic in the summer months.

**Evripos channel:** The area is located in the town of Chalkida (Euboea, Eastern Mediterranean) and is characterised by strong hydrodynamic changes caused by strong tidal currents. The midlittoral zone of this channel is an artificial hard bottom habitat (concrete). Three stations were chosen in this area with different levels of hydrodynamism: Evripos_1a with low, Evripos_1b with moderate and Evripos_1c with high hydrodynamic intensity. Evripos_1a is characterised by dense photophilous algal coverage dominated by *Corallina elongata*. Evripos_1b is covered by photophilous macroalgae (60%) and by the mollusk *Mytilus galloprovincialis* (40%). Finally, the station Evripos_1c is characterised by high densities of *Mytilus galloprovincialis*. Despite their urban location, the stations are not noticeably affected by organic discharges since the strong currents prevailing in the area dissipate pollution.

**Thermaikos Gulf:** Thermaikos Gulf is an embayment in the North part of the Aegean Sea (Eastern Mediterranean) and is strongly impacted by urban pollution. The midlittoral zone sampled here is an artificial hard bottom habitat (concrete). At this site, three stations were sampled, with an increase of pollution intensity from station Thermaikos_2a to Thermaikos_2c. The station Thermaikos_2a is located in Nea Mixaniona and is characterised by low hydrodynamic intensity. The algal coverage at this station is dominated by the macroalga *Antithamnion cruciatum*. The station Thermaikos_2b is located in Neoi Epivates and receives intense wave action. The substrate of this station is covered by beds of the mollusk *Mytilus galloprovincialis*. The station Thermaikos_2c is located in front of the Thessaloniki Concert Hall and is sheltered from strong waves. The substrate of this station is covered by the mollusk *Mytilus galloprovincialis* and the alga *Ulva lactuca*.

**Nea Roda and Porto Karas:** Both areas are located in Chalkidiki (North Aegean Sea, Eastern Mediterranean) but differ in terms of wave exposure: Nea Roda is moderately exposed, Porto Karas sheltered. The substrate in Nea Roda consists of granite, in Porto Karas the substrate is artificial (concrete). Mollusks are the dominant taxon in Nea Roda, whereas the midlittoral zone of Porto Karas is characterised by low densities of photophilous macroalgae. Nea Roda is a pristine area, whereas the stations in Porto Karas are located in a typical hotel marina and are subjected to slightly increased levels of organic pollution.

**Porto Lagos:** The sampling stations are located in a small port in Vistonicos Gulf (North Aegean Sea, Eastern Mediterranean) and is characterised by low-intensity hydrodynamism, low salinity and an artificial substrate (concrete). The midlittoral zone is dominated by the polychaete *Ficopomatus enigmaticus* which forms extensive biogenic calcareous layers of 3-4 cm height. Inside the port area, slightly increased levels of organic pollutions were detected.

**Balestrate and Zingaro:** Both areas are located in the Gulf of Castellammare. Balestrate is an outcrop of calcarenitic rocks surrounded by sand and is located in the centre of the Gulf. In this area, *Sabellaria alveolata* reefs temporarily proliferated between 1984–89 (preceding the sampling activities) in the infralittoral and midlittoral layers as a consequence of a wine distillery outfall. In the midlittoral zone, *Sabellaria alveolata* was associated with *Mytilaster* spp. beds. Zingaro, now a terrestrial and coastal reserve without influences from major anthropogenic stressors, is a steep calcareous cliff that stretches along the westernmost side of the Gulf. The midlittoral zone is characterised by the presence of vermetid reefs formed by the mollusk *Dendropoma petraeum*. Both areas are exposed to moderate wave action.

**Capo Gallo:** Capo Gallo, now a marine protected area, is a steep calcareous cliff located at the northern end of the Gulf of Palermo, not far from the city of Palermo. As in Zingaro, the midlittoral zone is characterised by the presence of vermetid reefs formed by the mollusk *Dendropoma petraeum*. The area is exposed to the dominant wind direction, resulting in increased wave action at the shore. No major sources of pollution are present in the vicinity.

## Sampling methods

### Study extent

**Figure 2.**
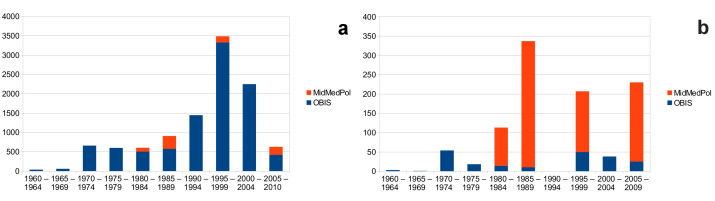
Temporal distribution of the number of polychaete records in the Mediterranean Sea present in the Ocean Biogeographic Information System (OBIS) and new contributions by the MidMedPol dataset. Only records from OBIS that contain information about the collection year were included, and data from before 1960 were omitted for reasons of clarity. **a** number of records for all depths, **b** number of records in the depth range of 0–5m. Diagrams based on the data from Suppl. material 1.

The data cover several independent sampling events over a time period of 25 years (1984–2009) and originate from 14 sampling sites in Italy and Greece (Mediterranean Sea). Samples were collected from the midlittoral zone from a maximum depth of 1.5 m. Concerning the distribution of polychaetes, this habitat is understudied in the Mediterranean Sea — in fact, the Ocean Biogeographic Information System contains less than 300 polychaete distribution records in the depth range of 0–5 m for the entire Mediterranean Sea, and none of these are from the intertidal zone. The present dataset thus provides an important addition to the exiting data for this habitat in the region (Fig. 2).

### Sampling description

Samples from Crete were collected from two sites, Alykes and Elounda. Both sites were sampled in September 2007, Alykes in June 2008 and Elounda in February 2009. Strong wave action prevented the site in Elounda from being sampled concurrently with the site in Alykes during the second year. Samples were collected according to the NaGISA protocol (Iken and Konar 2003). At each site, the high, mid- and low midlittoral zone was determined and five random replicate units were collected from each zone by placing a plexiglas frame (25x25 cm) on the substrate and scraping the framed area completely. The samples were then collected with a netted shovel into plastic bags, washed through a 0.5 mm mesh sieve and fixed in 99% ethanol. In the laboratory, all samples were identified to the most precise taxonomic level possible, using the most recent literature for the taxon. Animals without a head were considered as fragments and were not identified. The individual taxon counts were directly entered into electronic worksheets (Microsoft Excel), along with all metadata concerning the identification (date, identifier, notes, literature used). Thus, the introduction of additional errors during the transcription of lab notes into an electronic format was avoided.

Samples from Evripos channel, Thermaikos Gulf, Chalkidiki and Porto-Lagos were collected from September 1997 until October 1997. At each site, five random replicate units were collected. Two kind of samplers were used: (a) a metallic frame (20x20 cm) with a 0.5 mm mesh bag attached to its upper part (Chintiroglou and Koukouras 1992); (b) an iron frame (20x20 cm) with plastic threads woven through holes on the sides of the frame, forming a grid. The framed surface of the substrate was scraped and collected into plastic bags with 10% formalin. In the laboratory, the samples were washed through a 1.5 mm and a 0.5 mm mesh sieve and fixed in 5% formalin. All samples were sorted into major taxonomic groups and identified to species level using various identification keys, but only the polychaete species were digitised and included in the present dataset, in order to form a thematic entity. Data from the five replicates were pooled, the dataset for these records thus contains the average of abundances.

Samples from Italy were collected in 1984, 1986 and 1989. In Zingaro, samples were collected in spring of 1984, in Capo Gallo in spring, autumn and winter of 1986 and in Balestrate once per season in 1989. The number of replicate units per sample vary between 4 and 13. Samples were collected by scraping the surface of a 20x20 cm square, stored in plastic bags and subsequently fixed in a 5% solution of sea water and formalin.In the laboratory, samples were sieved through a 0.5 mm mesh size and preserved in 75% ethanol. Polychaetes were sorted into families and then identified to species level using various identification keys.

### Quality control

All scientific names were standardised against the World Register of Marine species using the Taxon Match tool (http://www.marinespecies.org/aphia.php?p=match). If recent taxonomic reviews were available that had not been incorporated into WoRMS at the time of standardisation, nomenclature follows those reviews. Subjective synonyms were kept in the dataset as they had been originally recorded, with a reference to the currently accepted name.

### Step description

**Figure 3.**
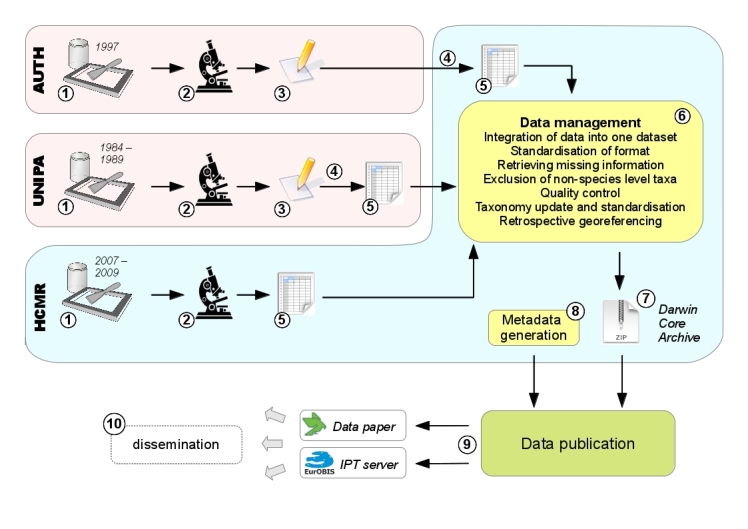
Overview of all steps leading to the final release of the dataset: **1** sampling, independently performed at the three different institutions (AUTH = Aristotle University of Thessaloniki, UNIPA = University of Palermo, HCMR = Hellenic Centre for Marine Research) **2** identification of polychaete specimens in the laboratory **3** data in paper-based format **4** digitisation **5** data in electronic format (spreadsheets) **6** integration of the three independent datasets into a standardised format, exclusion of records not identified to species level, retrieval of missing information, georeferencing of coordinates through Google Maps, standardisation of taxonomy against the World Register of Marine Species and recent literature, general quality control **7** export of data as a DarwinCore Archive **8** generation of dataset-level metadata **9** publication of the data as a data paper and through an IPT server **10** in the future, further dissemination of data by integration into other databases, personal downloads, archiving, etc.

The samples had been obtained independently by three different research teams over a period of 25 years as described in detail above. In an attempt to assemble polychaete occurrence data of the Mediterranean midlittoral zone, the datasets included in this study were obtained from the respective colleagues, cross-checked, annotated, quality-controlled and transformed into a standard electronic format (Fig. 3).

## Geographic coverage

### Description

Samples were collected at 14 sampling sites in Italy and Greece, Mediterranean Sea, from a maximum depth of 1.5 m (Table 1, Fig. 1). All data are collected from the midlittoral zone, characterised by the low and high water marks at those places where a tide is present, and the characteristics of the ecological zonation where the midlittoral zone is defined mainly by the gradient of emersion/ desiccation resulting from wave action.

The present dataset contains the first electronically available quantitative data on midlittoral polychaetes in the entire Mediterranean Sea. Previous studies of the habitat in the region are scarce, often qualitative and not electronically available.

### Coordinates

35.261249 and 41.005812 Latitude; 25.75173 and 12.8027 Longitude.

## Taxonomic coverage

### Description

**Kingdom**: Animalia

**Phylum**: Annelida

**Class**: Polychaeta

**Orders**: Sabellida, Terebellida, Eunicida, Phyllodocida, Amphinomida, Scolecida, Spionida

**Common names:** Bristle worms, segmented worms

The original dataset comprises distribution information for 123 polychaete species in 22 families. However, following recent taxonomic literature, several of the 127 species are currently regarded as synonyms. The present dataset, after updating the taxonomy, contains therefore distribution records for 123 species (Table 2). Of these, five species have been recorded for the first time in the respective area.

**Figure 4.**
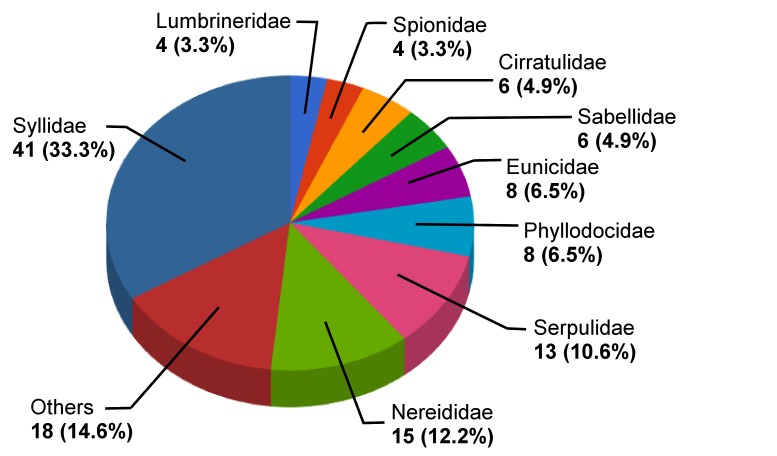
Distribution of species per family (abundance and percentage) for the most species-rich families in the MidMedPol dataset. Families with less than four species were combined in the category "Others". These are: Sabellariidae, Terebellidae (3 species each), Capitellidae (2 species), Amphinomidae, Aphroditidae, Chrysopetalidae, Dorvilleidae, Maldanidae, Oenonidae, Opheliidae, Orbiniidae, Pholoidae and Polynoidae (1 species each). Diagram based on the data from Suppl. material 2.

The species richness of the 22 families is very heterogeneous. Syllidae are the family with the highest species richness, comprising 33.3% of the species in the dataset, followed by Nereididae with 12.6% of the found species and Serpulidae with 10.6% (Fig. 4). Only nine families are represented by more than 3 species, whereas ten families are represented by a single species only.

**Figure 5.**
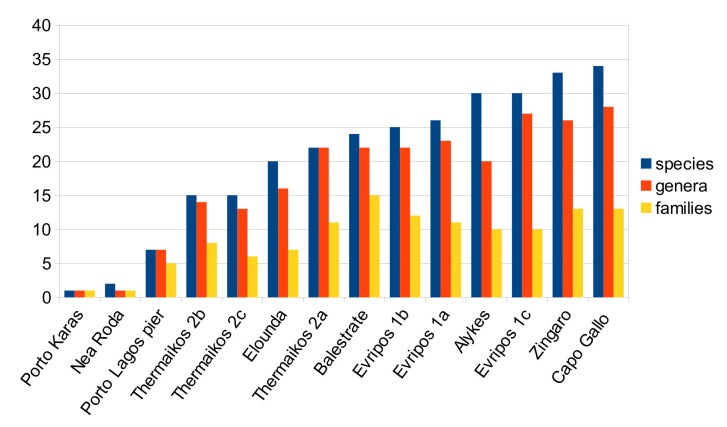
Number of species, genera and families per sampling location. Diagram based on the data from Suppl. material 3

Species richness at the different sampling sites is very heterogenous, with only a single species found in Porto Karas to 34 species found in Capo Gallo. Likewise, the number of higher taxa is different across locations, e.g. the 24 species recorded in Balestrate belong to 15 different families, whereas the 30 species recorded each in Alykes and Evripos St. 1c belong to only 10 families (Fig. 5).

## Temporal coverage

**Data range:** 1984 1 01 – 2009 5 20.

## Usage rights

### Use license

Open Data Commons Public Domain Dedication and License (PDDL)

### IP rights notes

The dataset can be freely used provided it is cited.

## Data resources

### Data package title

MidMedPol: Polychaetes from midlittoral rocky shores in Greece and Italy (Mediterranean Sea)

### Resource link

http://ipt.vliz.be/resource.do?r=mediterraneanpolychaetaintertidal

### Number of data sets

1

### Data set 1.

#### Data set name

MidMedPol: Polychaetes from midlittoral rocky shores in Greece and Italy (Mediterranean Sea)

#### Data format

Darwin Core Archive

#### Number of columns

51

#### Character set

UTF-8

#### Download URL

http://ipt.vliz.be/resource.do?r=mediterraneanpolychaetaintertidal

#### Data format version

1.0

#### Description

The dataset is available via the GBIF Internet Publishing Toolkit (IPT) of the Flanders Marine Institute (VLIZ). This IPT installation serves as the European node of the Ocean Biogeographic Information System (EurOBIS). The data will also be harvested by and made available through the International OBIS database, as well as through the data portal of the Global Biodiversity Information Facility (GBIF). The dataset is available as a DarwinCoreArchive, all fields are mapped to DarwinCore terms (http://rs.tdwg.org/dwc/).

This publication refers to the most recent version of the dataset available through the IPT server or EurOBIS. Future changes to the dataset due to quality control activities might change its content or structure.

**Data set 1.** 

Column label	Column description
recordNumber	A unique identifier for the record within the data set or collection.
scientificName	The scientific name of the taxon, including authorship.
scientificNameAuthorship	The authorship information for the scientificName formatted according to the conventions of the applicable nomenclaturalCode.
acceptedNameUsage	The full name, with authorship and date information if known, of the currently valid (zoological) taxon.
taxonRemarks	Comments or notes about the taxon or name.
specificEpithet	The species epithet of the scientificName.
identificationQualifier	A brief phrase or a standard term ("cf.", "aff.") to express the determiner's doubts about the Identification.
genus	The full scientific name of the genus in which the taxon is classified.
family	The full scientific name of the family in which the taxon is classified.
order	The full scientific name of the orde in which the taxon is classified.
class	The full scientific name of the class in which the taxon is classified.
phylum	The full scientific name of the phylum in which the taxon is classified.
kingdom	The full scientific name of the kingdom in which the taxon is classified.
fieldNumber	Denotes the code of each replicate unit.
fieldNotes	Notes about this occurrence record.
EventDate	The sampling date.
verbatimEventDate	The verbatim expression of the sampling date.
year	The sampling year.
month	The sampling month.
day	The sampling day.
locality	The specific location where the sample was taken.
municipality	The full, unabbreviated name of the next smaller administrative region than county (city, municipality, etc.) in which the sampling location occurs.
island	The name of the island on or near which the sampling location occurs.
country	The name of the country in which the sampling location occurs.
countryCode	The standard code of the country in which the sampling location occurs.
continent	The name of the continent in which the sampling location occurs.
waterBody	The name of the water body in which the sampling location occurs.
decimalLatitude	The geographic latitude (in decimal degrees, using the spatial reference system given in geodeticDatum) of the geographic center of a Location. Positive values are north of the Equator, negative values are south of it. Legal values lie between -90 and 90, inclusive.
decimalLongitude	The geographic longitude (in decimal degrees, using the spatial reference system given in geodeticDatum) of the geographic center of a Location. Positive values are east of the Greenwich Meridian, negative values are west of it. Legal values lie between -180 and 180, inclusive.
GeoreferenceSources	A list (concatenated and separated) of maps, gazetteers, or other resources used to georeference the Location, described specifically enough to allow anyone in the future to use the same resources.
coordinateUncertaintyInMeters	The horizontal distance (in meters) from the given decimalLatitude and decimalLongitude describing the smallest circle containing the whole of the sampling location.
habitat	A category or description of the habitat from which the samples were collected.
minimumDepthInMeters	The lesser depth of a range of depth below the local surface, in meters.
maximumDepthInMeters	The greater depth of a range of depth below the local surface, in meters.
samplingProtocol	The description of the method or protocol used for sample collection.
basisOfRecord	The specific nature of the data record, as described in http://rs.tdwg.org/dwc/terms/type-vocabulary/index.htm.
preparations	Preparations and preservation methods for a specimen.
individualCount	The number of individuals in a replicate sample unit. In cases where replicates had been pooled, the average abundances are not included under "individualCount" but under "dynamicProperties"
dynamicProperties	Includes here as the only attribute "meanAbundance". These are the average abundances of those samples where the replicates had been pooled.
recordedBy	A list (concatenated and separated) of names of people responsible for recording the original Occurrence.
identifiedBy	A list (concatenated and separated) of names of people, groups, or organizations who identified the specimen.
dateIdentified	The date on which the specimen was identified.
identificationReferences	A list (concatenated and separated) of references (publication, global unique identifier, URI) used for identifying the specimen.
institutionCode	The name (or acronym) in use by the institution having custody of the object(s) or information referred to in the record.
institutionID	An identifier for the institution having custody of the object(s) or information referred to in the record.
datasetID	An identifier for the set of data.
datasetName	The name identifying the data set from which the record was derived.
rights	Information about rights held in and over the resource (copyright, intellectual property, etc.).
rightsHolder	A person or organization owning or managing rights over the resource.
id	A unique identifier for the record within the data set or collection, auto-incrementing number automatically added by the system.
taxonID	Aphia ID (Unique Identifier for the taxon within the World Register of Marine Species - www.marinespecies.org)

## Additional information

### Resource citation

Keklikoglou, K., Faulwetter, S., Chatzigeorgiou, G., Badalamenti, F., Kitsos, MS., Arvanitidis, C. (2013). MidMedPol: Polychaetes from midlittoral rocky shores in Greece and Italy (Mediterranean Sea). 788 records, Contributed by Arvanitidis, C., Chatzigeorgiou, G., Faulwetter, S., Keklikoglou, K., Badalamenti, F., Kitsos, MS., Tyberghein, L., Plaiti, W., Markantonatou, V., Pesmatzoglou, I., Fernandez R. and students from Niceville High School, FL, USA and Ousantzopoulou K. and students from Heraklion High School of Arts, Crete, Greece, Online http://ipt.vliz.be/resource.do?r=mediterraneanpolychaetaintertidal, Version 1.0. Data Paper ID: doi: 10.3897/BDJ.1.e961

## Figures and Tables

**Table 1.** Coordinates, depth and sampling dates of the sampling localities

Country	Sampling Site	Latitude	Longitude	Minimum depth (in meters)	Maximum depth (in meters)	Sampling period
Greece	Alykes	35.41461	24.98816	0	0.5	09/2007 and 06/2008
Greece	Elounda	35.26125	25.75178	0	0.5	09/2007 and 05/2009
Greece	Evripos channel (St. 1a)	38.46432	23.5917	0	0.2	09/1997-10/1997
Greece	Evripos channel (St. 1b)	38.46342	23.59038	0	0.2	09/1997-10/1997
Greece	Evripos channel (St. 1c)	38.46302	23.58922	0	0.2	09/1997-10/1997
Greece	Thermaikos Gulf (St.2a)	40.46238	22.85345	0	0.2	09/1997-10/1997
Greece	Thermaikos Gulf (St.2b)	40.50664	22.9078	0	0.2	09/1997-10/1997
Greece	Thermaikos Gulf (St.2c)	40.59685	22.94731	0	0.2	09/1997-10/1997
Greece	Nea Roda	40.38238	23.93882	0	1.5	09/1997-10/1997
Greece	Porto Karas	40.07488	23.79555	0	0.3	09/1997-10/1997
Greece	Porto Lagos	41.00581	25.11961	0	0.2	09/1997-10/1997
Italy	Balestrate	38.05072	12.99988	0	0.3	1989 (spring, summer, autumn, winter)
Italy	Zingaro	38.09721	12.8027	0	0.3	1984 (spring)
Italy	Capo Gallo	38.21174	13.28836	0	0.3	1986 (spring, autumn, winter)

**Table 2.** Taxa identified to species-level are included in the dataset and new records for the geographic areas.

Family	Species	New record for area	References used for identification
Amphinomidae	*Chloeia venusta* Quatrefages, 1866		Fauvel 1923
Aphroditidae	*Pontogenia chrysocoma* (Baird, 1865)		Fauvel 1923
Capitellidae	*Capitella capitata* (Fabricius, 1780)		Fauvel 1927
Capitellidae	*Dasybranchus caducus* (Grube, 1846)		Fauvel 1927
Chrysopetalidae	*Chrysopetalum debile* (Grube, 1855)		Fauvel 1923, Vieitez et al. 2004
Cirratulidae	*Aphelochaeta filiformis* (Keferstein, 1862)		Fauvel 1927
Cirratulidae	*Aphelochaeta marioni* (de Saint Joseph, 1894)		Fauvel 1927
Cirratulidae	*Caulleriella alata* (Southern, 1914)		Fauvel 1927
Cirratulidae	*Cirriformia chrysoderma* (Claparède, 1869)		Fauvel 1927
Cirratulidae	*Dodecaceria concharum* Örsted, 1843		Fauvel 1927
Cirratulidae	*Timarete filigera* (Delle Chiaje, 1828)		Fauvel 1927
Dorvilleidae	*Schistomeringos rudolphii* (delle Chiaje, 1828)		Fauvel 1923
Eunicidae	*Eunice purpurea* Grube, 1866		Fauvel 1923
Eunicidae	*Eunice torquata* Quatrefages, 1866		Fauvel 1923
Eunicidae	*Lysidice collaris* Grube, 1870		Fauvel 1923, http://www.ceab.csic.es/~dani/Lysidice.html
Eunicidae	*Lysidice ninetta* Audouin & Milne-Edwards, 1833		Fauvel 1923, http://www.ceab.csic.es/~dani/Lysidice.html
Eunicidae	*Marphysa fallax* Marion & Bobretzky, 1875		Fauvel 1923
Eunicidae	*Marphysa sanguinea* (Montagu, 1815)		Fauvel 1923
Eunicidae	*Nematonereis unicornis* (Grube, 1840)		Fauvel 1923, Fauchald 1977
Eunicidae	*Palola siciliensis* (Grube, 1840)		Fauvel 1923
Lumbrineridae	*Lumbrineris coccinea* (Renier, 1804)		Carrera-Parra 2006
Lumbrineridae	*Lumbrineris inflata* Moore, 1911		Fauvel 1923
Lumbrineridae	*Scoletoma funchalensis* (Kinberg, 1865)		Fauvel 1923
Lumbrineridae	*Scoletoma impatiens* (Claparède, 1868)		Fauvel 1923
Maldanidae	*Praxillella gracilis* (M. Sars, 1861)		Fauvel 1927
Nereididae	*Ceratonereis costae* (Grube, 1840)		Fauvel 1923
Nereididae	*Neanthes caudata* (Delle Chiaje, 1827)		Fauvel 1923
Nereididae	*Neanthes fucata* (Savigny in Lamarck, 1818)	Aegean Sea, Greece	Vieitez et al. 2004
Nereididae	*Neanthes nubila* (Quatrefages, 1865)		Vieitez et al. 2004
Nereididae	*Nereis splendida* Grube, 1840		Fauvel 1923
Nereididae	*Nereis lamellosa* Ehlers, 1864		Vieitez et al. 2004
Nereididae	*Nereis pelagica* Linnaeus, 1758		Fauvel 1923
Nereididae	*Nereis perivisceralis* Claparède, 1868	Eastern Mediterranean	Vieitez et al. 2004
Nereididae	*Nereis pulsatoria* (Savigny, 1822)		Vieitez et al. 2004
Nereididae	*Nereis rava* Ehlers, 1864		Fauvel 1923
Nereididae	*Perinereis cultrifera* (Grube, 1840)		Fauvel 1923, Vieitez et al. 2004
Nereididae	*Perinereis macropus* (Claparède, 1870)		Fauvel 1923
Nereididae	*Platynereis dumerilii* (Audouin & Milne Edwards, 1834)		Fauvel 1923, Vieitez et al. 2004
Nereididae	*Pseudonereis anomala* Gravier, 1900		Vieitez et al. 2004
Nereididae	*Websterinereis glauca* (Claparède, 1870)		Fauvel 1923
Oenonidae	*Arabella geniculata* (Claparède, 1868)		Fauvel 1923
Opheliidae	*Polyophthalmus pictus* (Dujardin, 1839)		Fauvel 1927
Orbiniidae	*Protoaricia oerstedi* (Claparède, 1864)		Fauvel 1927
Pholoidae	*Pholoe inornata* Johnston, 1839		Barnich and Fiege 2003
Phyllodocidae	*Eulalia clavigera* (Audouin & Milne Edwards, 1833)	Aegean Sea, Greece	Vieitez et al. 2004
Phyllodocidae	*Eulalia viridis* (Linnaeus, 1767)		Fauvel 1923
Phyllodocidae	*Eumida sanguinea* (Örsted, 1843)		Vieitez et al. 2004
Phyllodocidae	*Mysta picta* (Quatrefages, 1865)		Vieitez et al. 2004
Phyllodocidae	*Nereiphylla rubiginosa* (Saint-Joseph, 1888)		Fauvel 1923
Phyllodocidae	*Phyllodoce macrophthalma* Schmarda, 1861		Fauvel 1923
Phyllodocidae	*Phyllodoce madeirensis* Langerhans, 1880		Fauvel 1923
Phyllodocidae	*Pterocirrus macroceros* (Grube, 1860)		Fauvel 1923
Polynoidae	*Lepidonotus clava* (Montagu, 1808)		Barnich and Fiege 2003, Fauvel 1923
Sabellariidae	*Sabellaria alcocki* Gravier, 1906		Fauvel 1927
Sabellariidae	*Sabellaria alveolata* (Linnaeus, 1767)		Fauvel 1927
Sabellariidae	*Sabellaria spinulosa* Leuckart, 1849		Fauvel 1927
Sabellidae	*Amphiglena mediterranea* (Leydig, 1851)		Fauvel 1927
Sabellidae	*Branchiomma lucullanum* (Delle Chiaje, 1828)		Fauvel 1927
Sabellidae	*Chone collaris* Langerhans, 1881		Fauvel 1927
Sabellidae	*Demonax brachychona* (Claparède, 1870)		Fauvel 1927
Sabellidae	*Oriopsis armandi* (Claparède, 1864)		Fauvel 1927
Sabellidae	*Pseudopotamilla reniformis* (Bruguière, 1789)		Fauvel 1927
Serpulidae	*Ficopomatus enigmaticus* (Fauvel, 1923)		Fauvel 1927
Serpulidae	*Hydroides dianthus* (Verrill, 1873)		Fauvel 1927
Serpulidae	*Hydroides elegans* (Haswell, 1883)		Fauvel 1927
Serpulidae	*Janua pagenstecheri* (Quatrefages, 1865)		Fauvel 1927
Serpulidae	*Pileolaria militaris* Claparède, 1868		Fauvel 1927
Serpulidae	*Serpula concharum* Langerhans, 1880		Fauvel 1927
Serpulidae	*Serpula vermicularis* Linnaeus, 1767		Fauvel 1927
Serpulidae	*Simplaria pseudomilitaris* (Thiriot-Quievreux, 1965)		Fauvel 1927
Serpulidae	*Spirobranchus lamarcki* (Quatrefages, 1866)		Fauvel 1927
Serpulidae	*Spirobranchus polytrema* (Philippi, 1844)		Fauvel 1927
Serpulidae	*Spirorbis marioni* Caullery & Mesnil, 1897		Fauvel 1927
Serpulidae	*Vermiliopsis infundibulum* (Philippi, 1844)		ten Hove and Kupriyanova 2009
Serpulidae	*Vermiliopsis striaticeps* (Grube, 1862)		Fauvel 1927
Spionidae	*Dipolydora armata* (Langerhans, 1880)		Fauvel 1927
Spionidae	*Dipolydora flava* (Claparède, 1870)		Fauvel 1927
Spionidae	*Polydora ciliata* (Johnston, 1838)		Fauvel 1927
Spionidae	*Polydora hoplura* Claparède, 1869		Fauvel 1927
Syllidae	*Branchiosyllis exilis* (Gravier, 1900)		San Martín 2003
Syllidae	*Brania pusilla* (Dujardin, 1851)		Fauvel 1923
Syllidae	*Exogone dispar* (Webster, 1879)		Fauvel 1923
Syllidae	*Haplosyllis spongicola* (Grube, 1855)		Fauvel 1923
Syllidae	*Myrianida convoluta* (Cognetti, 1953)		San Martín 2003
Syllidae	*Myrianida edwardsi* (Saint Joseph, 1887)		Fauvel 1923
Syllidae	*Myrianida prolifera* (O.F. Müller, 1788)		Fauvel 1923
Syllidae	*Myrianida quindecimdentata* (Langerhans, 1884)		San Martín 2003
Syllidae	*Odontosyllis ctenostoma* Claparède, 1868		Fauvel 1923
Syllidae	*Odontosyllis gibba* Claparède, 1863		Fauvel 1923
Syllidae	*Opisthosyllis brunnea* Langerhans, 1879		San Martín 2003
Syllidae	*Paraehlersia ferrugina* (Langerhans, 1881)		Fauvel 1923
Syllidae	*Prosphaerosyllis xarifae* (Hartmann-Schröder, 1960)		San Martín 2003
Syllidae	*Salvatoria clavata* (Claparède, 1863)		Fauvel 1923
Syllidae	*Salvatoria neapolitana* (Goodrich, 1930)		San Martín 2003
Syllidae	*Sphaerosyllis bulbosa* Southern, 1914		Fauvel 1923
Syllidae	*Sphaerosyllis hystrix* Claparède, 1863		Fauvel 1923
Syllidae	*Sphaerosyllis ovigera* Langerhans, 1879		Fauvel 1923
Syllidae	*Sphaerosyllis pirifera* Claparède, 1868		Fauvel 1923, San Martín 2003
Syllidae	*Syllis amica* Quatrefages, 1866		Fauvel 1923, San Martín 2003
Syllidae	*Syllis armillaris* (O.F. Müller, 1776)		Fauvel 1923, San Martín 2003
Syllidae	*Syllis beneliahuae* (Campoy & Alquézar, 1982)		San Martín 2003
Syllidae	*Syllis cf mayeri*. Musco & Giangrande, 2005	Aegean Sea, Greece	Aguado and San Martín 2007
Syllidae	*Syllis columbretensis* (Campoy, 1982)		San Martín 2003
Syllidae	*Syllis compacta* Gravier, 1900		San Martín 2003
Syllidae	*Syllis corallicola* Verrill, 1900		San Martín 2003
Syllidae	*Syllis cornuta* Rathke, 1843		Fauvel 1923
Syllidae	*Syllis garciai* (Campoy, 1982)		San Martín 2003
Syllidae	*Syllis gerlachi* (Hartmann-Schröder, 1960)		San Martín 2003
Syllidae	*Syllis golfonovensis* (Hartmann-Schröder, 1962)		San Martín 1984
Syllidae	*Syllis gracilis* Grube, 1840		Fauvel 1923, San Martín 2003
Syllidae	*Syllis hyalina* Grube, 1863		Fauvel 1923, San Martín 2003
Syllidae	*Syllis kabilica* Ben-Eliahu, 1977	Italy	San Martín 1984
Syllidae	*Syllis krohni* Ehlers, 1864		Fauvel 1923, San Martín 2003
Syllidae	*Syllis prolifera* Krohn, 1852		Fauvel 1923
Syllidae	*Syllis rosea* (Langerhans, 1879)		Fauvel 1923, San Martín 2003
Syllidae	*Syllis variegata* Grube, 1860		Fauvel 1923
Syllidae	*Syllis vittata* Grube, 1840		Fauvel 1923
Syllidae	*Syllis westheidei* San Martín, 1984		San Martín 2003
Syllidae	*Trypanosyllis coeliaca* Claparède, 1868		Fauvel 1923
Syllidae	*Trypanosyllis zebra* (Grube, 1840)		Fauvel 1923, San Martín 2003
Terebellidae	*Nicolea venustula* (Montagu, 1818)		Holthe 1986
Terebellidae	*Amphitritides gracilis* (Grube, 1860)		Fauvel 1927
Terebellidae	*Terebella lapidaria* Linnaeus, 1767		Fauvel 1927

## Supplementary files

### Number of polychaete records in the Mediterranean from OBIS

**Authors:** Sarah Faulwetter **Data type:** Microsoft Excel file Number of polychaete records in the Mediterranean in five-year intervals since 1960. Data from the Ocean Biogeographic information system, plus additions from the present dataset. **File:** oo_4274.xls

### Number of species per family

**Authors:** Kleoniki Keklikoglou **Data type:** Microsoft Excel spreadsheet Summary of the number of species per family. **File:** oo_4271.xls

### Number of species, genera and families per sampling station

**Authors:** Sarah Faulwetter **Data type:** Microsoft Excel spreadsheet Overview of the number of taxa per sampling station **File:** oo_4273.xls
